# Tissue Source and Cell Expansion Condition Influence Phenotypic Changes of Adipose-Derived Stem Cells

**DOI:** 10.1155/2017/7108458

**Published:** 2017-08-23

**Authors:** Lauren H. Mangum, Shanmugasundaram Natesan, Randolph Stone, Nicole L. Wrice, David A. Larson, Kyle F. Florell, Barbara A. Christy, Maryanne C. Herzig, Andrew P. Cap, Robert J. Christy

**Affiliations:** ^1^Combat Trauma and Burn Injury Research, US Army Institute of Surgical Research, San Antonio Military Medical Center, JBSA Ft Sam Houston, San Antonio, TX, USA; ^2^Coagulation and Blood Research, US Army Institute of Surgical Research, San Antonio Military Medical Center, JBSA Ft Sam Houston, San Antonio, TX, USA

## Abstract

Stem cells derived from the subcutaneous adipose tissue of debrided burned skin represent an appealing source of adipose-derived stem cells (ASCs) for regenerative medicine. Traditional tissue culture uses fetal bovine serum (FBS), which complicates utilization of ASCs in human medicine. Human platelet lysate (hPL) is one potential xeno-free, alternative supplement for use in ASC culture. In this study, adipogenic and osteogenic differentiation in media supplemented with 10% FBS or 10% hPL was compared in human ASCs derived from abdominoplasty (HAP) or from adipose associated with debrided burned skin (BH). Most (95–99%) cells cultured in FBS were stained positive for CD73, CD90, CD105, and CD142. FBS supplementation was associated with increased triglyceride content and expression of adipogenic genes. Culture in hPL significantly decreased surface staining of CD105 by 31% and 48% and CD142 by 27% and 35% in HAP and BH, respectively (*p* < 0.05). Culture of BH-ASCs in hPL also increased expression of markers of osteogenesis and increased ALP activity. These data indicate that application of ASCs for wound healing may be influenced by ASC source as well as culture conditions used to expand them. As such, these factors must be taken into consideration before ASCs are used for regenerative purposes.

## 1. Introduction

Adipose tissue is a rich reservoir of mesenchymal stem cells (ASCs) with a high self-renewing capacity [1–4]. ASCs are immune-privileged, multipotent cells that have been extensively investigated as a treatment option for various pathological and traumatic conditions [5–9]. ASCs have the resiliency to adapt to austere host conditions and have been shown to benefit healing through direct cellular interactions or by paracrine signaling mechanisms [10–13]. Due to the unique ability of ASCs to elicit this beneficial healing response, clinical studies have been conducted using autologous stem cells for regenerative therapies [14–18]. Prior to performing a preclinical or clinical study, ASCs should be characterized for their immunophenotype, proliferation, and multilineage differentiation potential. According to the International Society of Cellular Therapy (ISCT) and International Federation of Adipose Therapeutics and Sciences (IFATS) panel of experts, freshly isolated stromal vascular fraction, as well as culture-expanded stem cells, are expected to express a positive panel of surface markers—CD13, CD73, CD90, and CD105—and remain negative for markers like CD31 and CD45. Furthermore, the stem cells must be checked for their ability to functionally differentiate into osteogenic, adipogenic, and chondrogenic lineages [19].

While immunophenotypic characteristics of the ASCs are well established, studies have shown some subtle differences in stem cell phenotype and differentiation capacity at least partly based on the influence of *in vitro* culture conditions [20, 21]. In addition, due to the rising concerns of possible antigenicity of FBS or its ability to potentially transmit zoonoses like prion diseases that may elude testing, alternative media compositions are under investigation [22, 23]. One such culture condition utilizes hPL as a growth supplement [24]. ASCs expanded in media supplemented with hPL *in lieu* of FBS have been shown to retain stem cell phenotype and multilineage differentiation capacity [25]. However, some recent reports have raised concerns that hPL might compromise the immunomodulatory and differentiation capacity of ASCs [26].

Another facet in the transition of ASCs into the clinical setting requires establishing the safety and stemness of the cells isolated from a patient's own body. Although allogeneic ASCs are an attractive off-the-shelf option due their availability and inherent immunomodulatory effect, clinical studies using ASCs have largely been limited to autologous treatment [6, 8, 16–18]. Considering the regulatory concerns regarding allogenic stem cells and any possible adverse effects, the Food and Drug Administration (FDA) has yet to establish the therapeutic classification of ASCs. Currently, only autologous ASCs are approved for use in regenerative therapies. Recently, we have established a method to isolate ASCs from the subcutaneous adipose tissue of burn patients' debrided skin and have shown that these stem cells do retain “stemness” and multilineage differentiation potential on par with ASCs derived from uninjured skin [27]. The isolated stem cells from the discarded skin were positive for a panel of stem cell makers including CD54, CD71, CD90, CD105, STRO-1, and PDGFR*β* and negative for CD45. Furthermore, we were able to show that these cells could be successfully transplanted to an athymic rat excision wound and demonstrate successful engraftment [27]. While our results showed feasibility of isolating ASCs in considerable quantities from burn patients and that these cells could be successfully engrafted into a wound bed, more investigation is required to establish the stem cell quality when expanded in xeno-free media. Therefore, the current study investigates how media supplemented with hPL, compared to FBS, affect the differentiation ability of ASCs isolated from either the subcutaneous adipose tissue from debrided burned tissue or uninjured adipose tissue derived from individuals undergoing abdominoplasty procedure.

## 2. Methods

### 2.1. Quantification of Chemokines in Human Platelet Lysate

Three lots of good manufacturing practice (GMP) grade hPL were purchased from Cook Medical (Stemulate; Cook Regentec, Indianapolis, IN). The concentration of analytes in the hPL was determined using the Quantibody® Multiplex enzyme-linked immunosorbent assay (ELISA) array (Q4000; RayBiotech, Norcross, GA) according to manufacturer's instructions. Briefly, the glass slides provided by RayBiotech were allowed to equilibrate to room temperature (RT) and air dry for 2 hours. Wells were blocked with sample diluent for 30 min at RT on a rocker. Standards were prepared as indicated while samples were diluted 1 : 1 in diluent buffer and incubated overnight at 4°C with mild shaking. Slides were washed, incubated with biotinylated antibody cocktail at RT for 2 hours, washed, incubated with Cy3 equivalent dye-streptavidin at RT for 1 hour, washed, dried, and read on an InnoScan 710 microarray scanner (Carbonne, France). Data was extracted and processed using the supplied Quantibody Q-Analyzer software. Standard curves were created for each analyte. The median fluorescent signal (532 nm) for each analyte was adjusted to background intensity, and the concentration was determined based on its respective standard curve. The concentration of each analyte was averaged for the three lots of hPL.

### 2.2. Isolation and Culture of ASCs

Samples used in the current experiments were cryopreserved isolates collected during a previous study by our laboratory [27]. These isolates were collected from the subcutaneous adipose tissue of discarded, debrided burned skin from patients undergoing tangential excision following burn trauma or were isolated from subcutaneous adipose tissue from discarded skin originating from abdominoplasty surgery in accordance with protocols reviewed and approved by the U.S. Army Medical Research and Materiel Command Institutional Review Board (H-11-020/M-10128). Throughout the manuscript, the following terms will be used to describe the ASC source:

- BH (burned human) refers to the ASCs derived from the subcutaneous fat remaining on the debrided burned skin samples.
- HAP (human abdominoplasty) refers to the ASCs derived from the adipose tissue harvested from skin samples of patients undergoing abdominoplasty procedure.

A total of 6 donors were used for isolates generated in our labs; each isolate was derived from a unique donor. For all experiments, three isolates per group (*n* = 3 BH and *n* = 3 HAP) were used, with at least three technical replicates per isolate per experiment. Briefly, the subcutaneous adipose tissue was separated from the skin samples and finely minced in Hanks' balanced salt solution (HBSS; Invitrogen, Carlsbad, CA) with sterile scissors. Samples were centrifuged and the resulting fatty layer was digested on an orbital shaker in HBSS with collagenase II (Life Technologies, Carlsbad, CA) to a final concentration of 1 mg/ml at 37°C. The digested cell suspension was filtered using 100 and 70 *μ*m mesh filters. The resulting ASCs were plated, expanded in MesenPRO RS media, and transferred to liquid nitrogen for long-term storage. A commercially available adipose-derived stem cell isolate, derived from a healthy individual, was purchased from Rooster Bio (Rooster Bio, Fredrick, MD) and used as a control group for all experiments. These cells, referred to in this manuscript as commercial ASCs (or RB), were expanded in a commercial media provided by the vendor (High Performance Media Kit, Rooster Bio), and switched to induction or control media containing FBS for all differentiation experiments.

### 2.3. Immunophenotyping by FACS

Three isolates of cryopreserved BH and HAP ASCs were resuspended in low-glucose Dulbecco's modified Eagle's medium (Gibco® DMEM; Thermo Fisher Scientific, Frederick, MD) supplemented with either 10% FBS (GE Life Sciences; Logan, Utah) or 10% hPL. Cells were expanded to passage 2 or 3 (P2 or P3), trypsinized, washed, resuspended at 1 × 10^6^ cells/ml in HBSS, stained with mouse anti-human CD44, CD73, CD90, CD105, and CD142 (Human MSC Analysis Kit, BD Biosciences, San Jose, CA), and analyzed by fluorescence-activated cell sorting (FACS). 1 × 10^5^ cells were incubated with 1% BSA and 5 *μ*l human Fc receptor blocking solution (BioLegend, San Diego, CA) for 5 min at room temperature (RT). Antibodies were added followed by 15 min of incubation at RT. Cells were then washed once in BD FACS Lyse Wash Assistant and analyzed on BD FACSCanto.

### 2.4. Cell Proliferation in hPL or FBS

Three isolates of cryopreserved BH and HAP ASCs were resuspended and cultured as described above. At P2 or P3, cells were trypsinized, resuspended in DMEM with either 10% FBS or hPL, seeded in black 96-well plates (BD Falcon, NJ) at a concentration of 2000 cells/well, and incubated for 1 to 7 days before analysis by CyQUANT Cell Proliferation Assay Kit (Molecular Probes, Eugene, OR) according to manufacturer's directions. Briefly, at each time point the media was removed, the plates were wrapped in parafilm and frozen at −80°C. After the final time point was collected, the plates were thawed at RT and lysed in 200 *μ*L of the CyQUANT GR dye/cell-lysis buffer (2x dye). Plates were incubated for 2–5 min in the dark at RT. Fluorescence was measured at an excitation of 485 nm and emission of 525 nm. Fluorescence was quantitated using a cell dilution series of 1000–64,000 cells/well. Growth curves were plotted as fluorescence versus time.

### 2.5. Adipogenic and Osteogenic Differentiation

Three isolates of cryopreserved BH and HAP ASCs were resuspended in culture media as described above. Cells were expanded to P2 or P3 in their respective media formulation before they were seeded in T-25 flasks (BD Falcon, NJ) and were allowed to reach near confluence (~90%) before induction towards the adipogenic or osteogenic lineages. Adipogenic differentiation media was composed of high-glucose (4.5 g/l) DMEM supplemented with 10% FBS or 10% hPL, 1 *μ*M dexamethasone (Sigma-Aldrich, St. Louis, MO), 200 *μ*M indomethacin (Sigma-Aldrich), 10 *μ*M insulin (Sigma-Aldrich), 0.5 *μ*M isobutylmethylxanthine (IBMX; Sigma-Aldrich), 10 *μ*M ciglitazone (Sigma-Aldrich), 2 mM L-glutamine (Life Technologies, Carlsbad, CA), and 100 IU penicillin and 100 *μ*g/ml streptomycin (Life Technologies). Cells were cultured in a 5% CO_2_-humidified incubator at 37°C for 14 days. To observe the staining of neutral lipids, cultures were rinsed with HBSS, fixed with 4% paraformaldehyde (PFA; Sigma-Aldrich) solution, and stained with Oil red O (Sigma-Aldrich). For osteogenic differentiation, cells from different patients were cultured to near confluence and induced with osteogenic differentiation media composed of *α*-MEM supplemented with 10% FBS or 10% hPL, 0.1 *μ*M dexamethasone, 50 *μ*M ascorbate-2-phosphate (Sigma Aldrich), 10 mM *β*-glycerophosphate (Sigma Aldrich), 10 ng/ml bone morphogenic protein-2 (BMP-2; R&D Systems, Minneapolis, MN), 2 mM L-glutamine, and 1% antibiotic-antimycotic. Induced cells were cultured in a 5% CO_2_-humidified incubator at 37°C for 7–14 days while cells cultured under identical conditions without induction factors served as undifferentiated, media controls.

### 2.6. Quantification of Adipogenesis and Osteogenesis

To observe the staining of neutral lipids, cultures were rinsed with HBSS, fixed with 4% PFA solution, and stained with Oil red O as previously described [28, 29]. Lipid accumulation was used as a marker of adipogenic differentiation and was assessed through quantitation of triglyceride within the cell. Briefly, cells were scraped in triglyceride assay (TG; Cayman Chemical, Ann Arbor, Michigan) lysis buffer, sonicated on ice, and centrifuged to pellet the cellular debris, and the resulting supernatant was used for triglyceride quantification using a TG assay according to manufacturer's instructions. Triglyceride measurements were normalized to total protein concentration using a bicinchoninic acid assay (BCA; Pierce, Rockford, IL). To assess mineralization, osteogenic cultures were rinsed with PBS and fixed with 10% neutral buffered formalin solution and calcium phosphate deposition was stained with 1% solution of Alizarin red S (pH 4.2, Sigma-Aldrich). Intracellular ALP (BioVision, Milpitas, CA) activity was quantified as a measure of mineralization and osteogenic differentiation. Control and osteogenic cultures were scraped in ice cold ALP buffer on day 7 and 14 postinduction. Samples were sonicated on ice and centrifuged to pellet insoluble debris. The resulting supernatant was diluted 1 : 10 or 1 : 20 in assay buffer, and ALP activity over 60 min was measured according to manufacturer's directions. ALP activity was normalized to total cellular protein content and reported as U/mg.

### 2.7. RNA Isolation and RT-PCR

Total RNA from differentiated cells was isolated using TRIzol LS reagent (TRIzol, Thermo Fisher). Cells were rinsed with HBSS and scraped into 1 ml TRIzol LS and incubated for 10–15 min in ice. Following incubation, 400 *μ*l of chloroform was added, mixed, and the aqueous phase separated by centrifugation. The RNA was then purified using Qiagen RNeasy Mini (Qiagen, Valencia, CA) spin columns. The concentration of RNA was determined at OD_260/280_ using a NanoDrop 8000 spectrometer (NanoDrop Technologies Inc., Wilmington, DE). Complementary DNA (cDNA) was synthesized using 1 *μ*g of total RNA to begin first-strand synthesis (VILO Superscript, Life Technologies, Carlsbad, CA). The following primer sets were used to evaluate the mRNA expression of specific markers (Thermo Fisher) for adipogenesis: adiponectin (*ADIPOQ*; forward: CGCTTGAGTTTAGGGTAACTGTGAAAGCG; reverse: GCTCACAGTCTCACATCTGGTTG), glucose transporter 4 (*GLUT4*; forward: CGATGCCAGCACTCCAGAAACATCG; reverse: GGGCCTGCCAGAAAGAGTCTG), leptin (*LEP*; forward: GGCTTTGGCCCTATCTTTTC; reverse: GCTCTTAGAGAAGGCCAGCA), peroxisome proliferator-activated receptor gamma (*PPARG*; forward: GCTCTAGAATGACCATGGTTGAC; reverse: ATAAGGTGGAGATGCAGGCTC); and osteogenesis: alkaline phosphatase (*ALPL*; SABiosciences, Germanton, MD), bone gamma-carboxyglutamate protein (*BGLAP*; SABiosiences), runt-related transcription factor 2 (*RUNX2*; SABiosiences), transcription factor Sp7 (*SP7*; SABiosiences), and secreted phosphoprotein 1 (*SPP1*; SABiosiences). Master mixes with 200 nM of forward/reverse primers and SYBR® Select Master Mix for CFX (Life Technologies, Carlsbad, CA) and 15 ng cDNA template were analyzed by RT-PCR using a Bio-Rad CFX96 thermal cycler system (Bio-Rad, Hercules, CA). Nontemplate control (NTC) and no reverse transcriptase (NRT) controls were run for each reaction. Gene expression was normalized to *β*-actin (*ACTB*; SABiosiences), and the fold change in expression levels for each mRNA transcript was determined by the 2^−ΔΔCT^ method [30]. Due to the lack of C_T_ values in the undifferentiated controls for *ADIPOQ*, *GLUT4*, *AND LEP*, all expression data from adipogenic conditions were normalized to commercial ASC adipogenic samples. For osteogenic differentiation and media control groups, C_T_ values were normalized against the noninduced RB cells in FBS-supplemented media to assess baseline expression. In order to account for isolate variability, it was determined that the C_T_ values for all HAP isolates and BH isolates would be averaged into one group/treatment condition and expressed as one data point, rather than as individual isolate numbers.

### 2.8. Statistical Analysis

For proliferation assays, statistical significance at each day was assessed by two-way ANOVA with a Sidak correction for multiple comparisons, and significance is designated with an asterisk. For all other experiments, statistically significant differences were determined with one-way ANOVA with Tukey's range test for multiple comparisons using GraphPad Prism 7.01 (GraphPad Software Inc., La Jolla, CA). For all figures, significant differences between groups are designated by alphabetical letters. Groups marked with the same letter are not statistically different from each other (*p* < 0.05, Tukey's MCT).

## 3. Results

### 3.1. Human Platelet Lysate Contains Detectable Levels of Cytokines and Growth Factors

**Figure 1:**
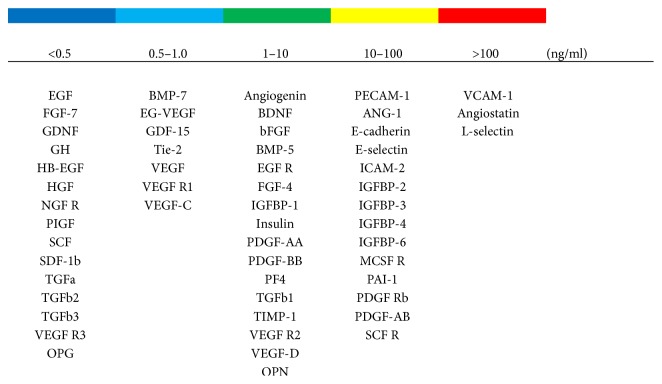
Quantification of cytokines, chemokines, and growth factors in human platelet lysate. Analysis of 200 proteins from three lots of human platelet lysate purchased from Cook Medical by Q4000 Quantibody revealed detectable levels of 186 proteins. A selection of proteins and growth factors related to growth, expansion, and differentiation are presented in ng/ml.

Protein concentrations of 200 cytokine, chemokine, and growth factors on three lots of human platelet lysate were analyzed by ELISA. Proteins of interest to the expansion, maintenance, and differentiation of adipose-derived stem cells were detected within the platelet lysate (Figure 1). Related to the growth and expansion of stem cells, platelet-derived growth factors (PDGF-AA, PDGF-AB, and PDGF-BB) were detected at 4567 pg/ml, 17,841 pg/ml, and 5238 pg/ml, respectively, while insulin-like growth factor-binding protein (IGFBP-2) was detectable at a concentration of 13,114 pg/ml. Insulin, which is added to adipogenic differentiation media to drive the differentiation process, was detectable at an average concentration of 2149 pg/ml. Relevant to osteogenic differentiation, osteopontin (OPN) and osteoprotegerin (OPG) were detected at an average of 164 pg/ml and 7369 pg/ml, respectively, while bone morphogenic proteins (BMP-5 and BMP-7) were detected on average at a concentration of 7238 pg/ml and 750 pg/ml, respectively.

### 3.2. Human Platelet Lysate Enhances Proliferation

**Figure 2:**
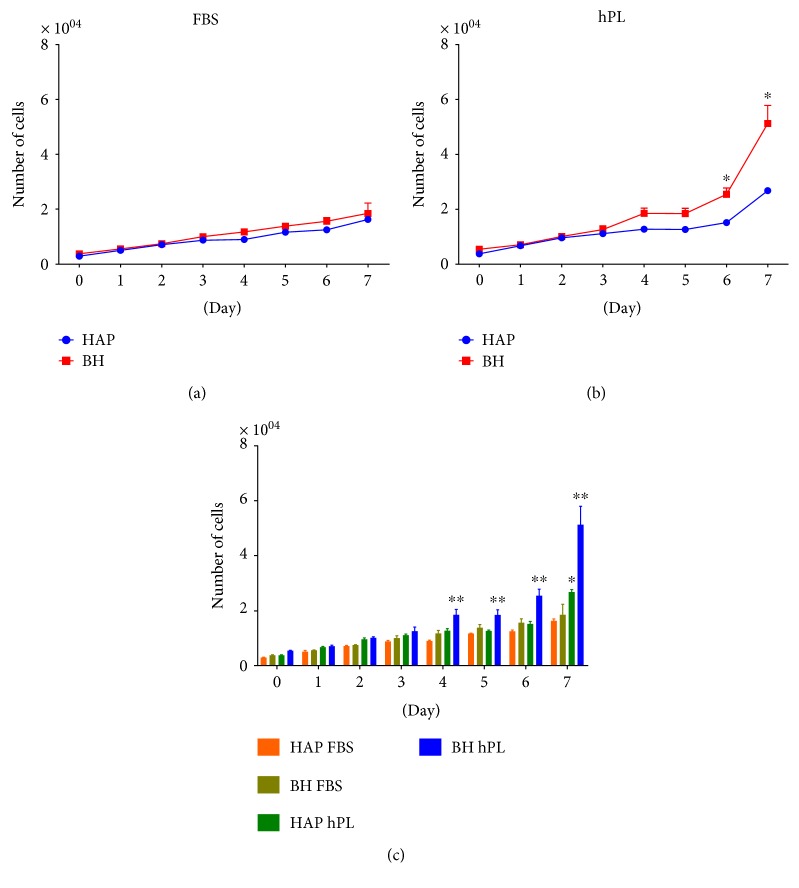
Proliferation of cells in FBS or hPL. Cells at P2 or P3 were seeded in a microplate at 2000 cells/well in media supplemented with FBS (a) or hPL (b) and allowed to proliferate for up to 7 days, with time points collected every 24 hrs. Data represents the mean ± SEM of three biological replicates from three donors/group. Statistically significant differences were determined by two-way ANOVA with Sidak's multiple comparison test. Significant differences between time points are designated by a single asterisk (b). When more than two groups are compared (c), columns with an asterisk (∗∗ or ∗) indicate significant differences from the remaining groups on a given day, while columns with different symbols are significantly different from each other (*p* < 0.05, Sidak's MCT).

Cell proliferation was measured over 7 days by CyQUANT Cell Proliferation Assay Kit. No significant differences were found between the average cell counts from BH or HAP groups at any time point for cells expanded in FBS-supplemented media (Figure 2(a) and Supplemental Figure 2A available online at https://doi.org/10.1155/2017/7108458). In hPL-supplemented media (Figure 2(b) and Supplemental Figure 2B), the average number of cells/well was significantly higher in BH isolates, compared to that in HAP isolates, at days 6 and 7. The mean number of RB cells/well was significantly higher from all BH and HAP isolates by day 3 and remained significantly higher until the end of the experiment (Supplemental Figure 2C).

### 3.3. Platelet Lysate Alters Cellular Surface Markers

**Figure 3:**
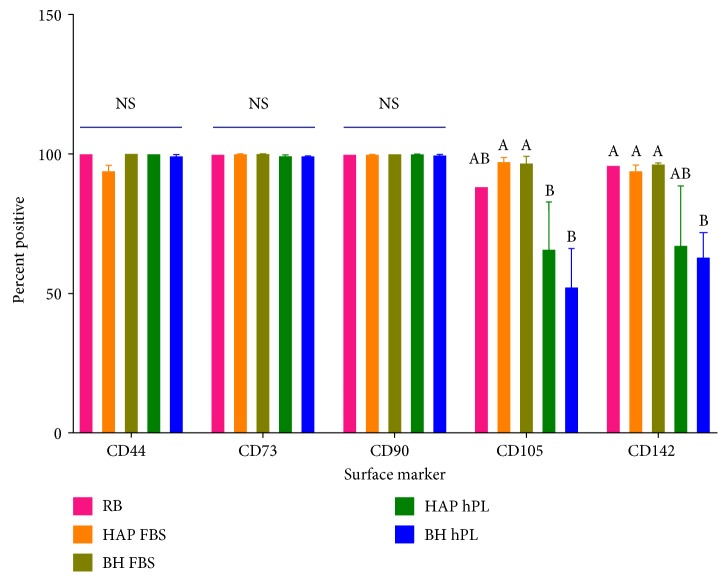
Effect of culture supplement on stemness phenotype. Cryopreserved HAP or BH cells were expanded to P2 or P3 in media supplemented with FBS or hPL. Data represents the mean ± SEM of three biological replicates from three donors/group. RB represents the mean ± SEM of three biological replicates from one donor from a commercial source. Significant differences between groups are designated by alphabetical letters. Any groups with the same letter are not statistically different from each other (*p* < 0.05, Tukey's MCT).

Expression of surface markers for stemness was evaluated by RT-PCR and FACS. Preliminary experiments indicated that there were no significant differences in transcript levels of the genes encoding CD73, CD90, and CD105 (Supplemental Figure 1), regardless of cell source or media supplement. PCR data was supported by FACS analysis, for cells expanded to P2 or P3 in FBS, as markers for stemness (CD44, CD73, CD90, and CD105), and tissue factors (CD142) were not significantly altered when compared to those of Rooster Bio ASCs. However, when cells were expanded in the presence of hPL, CD105 was significantly reduced in HAP and BH isolates as compared to their FBS counterparts. CD142 was reduced in HAP and BH cells cultured in hPL, but this reduction was only significant in the BH isolates (Figure 3()). Despite the differential surface staining of CD142, all isolates exhibited reduced clotting times (Supplemental Figure 3).

### 3.4. Differential Induction of Adipogenic Markers in Cells Grown in FBS or hPL

**Figure 4:**
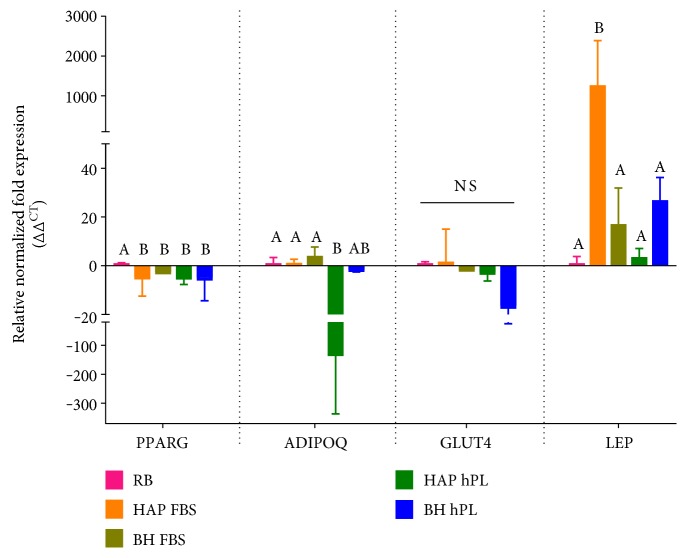
Expression of markers of adipogenesis. RNA was isolated from cells differentiated in media supplemented with 10% FBS or 10% hPL, and expression of *PPARG*, *ADIPOQ*, *GLUT4*, and *LEP* was measured by RT-PCR. Statistically significant differences were determined by one-way ANOVA with Tukey's multiple comparisons. Data represents the mean ± SEM of three biological replicates from three donors/group. RB represents the mean ± SEM of three biological replicates from one donor from a commercial source. Significant differences between groups are designated by alphabetical letters. Any groups with the same letter are not statistically different from each other (*p* < 0.05, Tukey's MCT).

Expression levels of markers of adipogenic differentiation were analyzed by RT-PCR (Figure 4). For undifferentiated conditions, only *PPARG* was expressed at levels high enough to generate a detectable C_T_. After adipogenic induction, *PPARG* was expressed at the highest level by the commercial ASCs (RB) compared to that by the other samples. A decrease in *ADIPOQ* expression was observed by the HAP-hPL and BH-hPL groups; however, the FBS-supplemented conditions were not different from the control. Though *GLUT4* expression levels were lower in the hPL-supplemented samples, the decrease in *GLUT4* expression was not significant. Lastly, *LEP* expression was higher in the HAP-FBS group and while the remaining groups were all slightly higher than commercial ASCs, any increase in expression did not achieve significance.

### 3.5. Lipid Accumulation Is Lower in Differentiated ASCs Grown in Platelet Lysate Media

**Figure 5:**
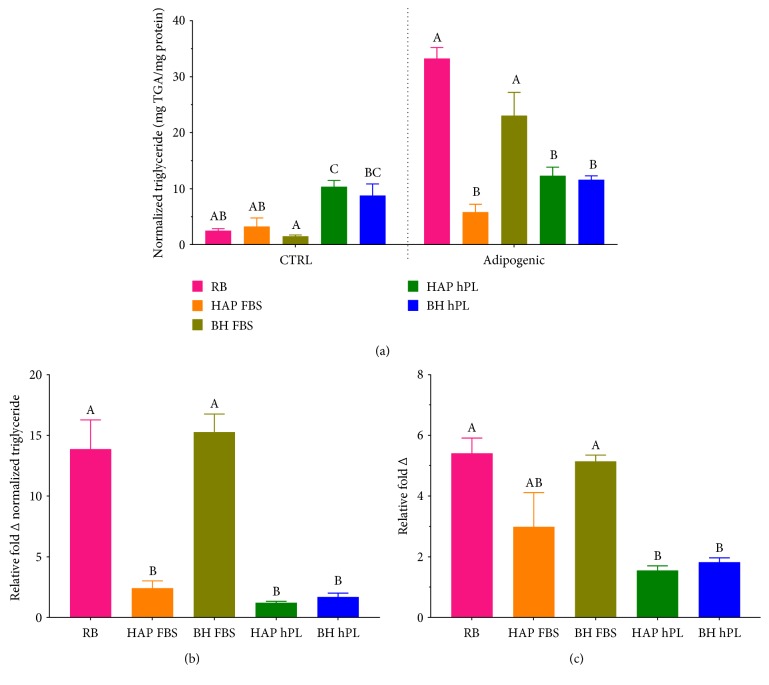
Assessment of lipid content of differentiated and undifferentiated ASCs. Triglyceride content and Oil red O were assessed in cells differentiated in media supplemented with 10% FBS or 10% hPL and normalized to total cellular protein content. Data are expressed as triglyceride content (mg/mg protein) in undifferentiated (CTRL) and differentiated (adipogenic) cells (a) and as fold change following adipogenic differentiation (b). Oil red O content is expressed as a relative fold change following adipogenic differentiation (c). Statistically significant differences were determined by one-way ANOVA with Tukey's multiple comparisons. Data represents the mean ± SEM of three biological replicates from three donors/group. RB represents the mean ± SEM of three biological replicates from one donor from a commercial source. Significant differences between groups are designated by alphabetical letters. Any groups with the same letter are not statistically different from each other (*p* < 0.05, Tukey's MCT).

**Figure 6:**
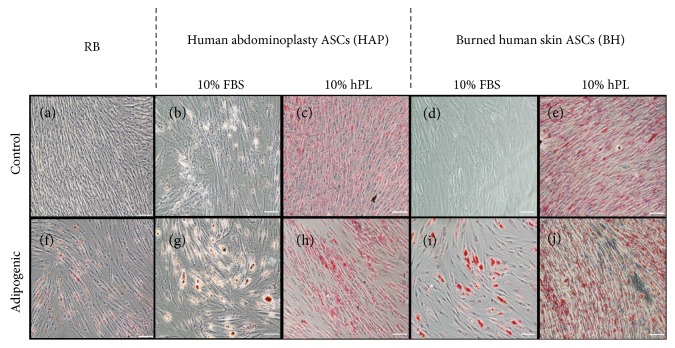
Light micrographs of Oil red O staining. ASCs bought from a commercial vendor (RB) were compared to ASCs derived from abdominoplasty (HAP) or burned human skin (BH) cultured in DMEM with 10% FBS and 10% hPL and differentiated under adipogenic conditions with identical supplements for 14 days, then stained for lipid accumulation by Oil red O. Representative images of undifferentiated RB (a), HAP cells (b, c), and BH cells (d, e) from control media conditions and differentiated RB (f), HAP cells (g, h), and BH (i, j) are given. White scale bar represents 100 *μ*m.

Triglyceride accumulation in undifferentiated (CTRL) and adipogenic-differentiated samples was assessed by commercial triglyceride assay and normalized to protein content of the cellular lysate. Results indicate that in undifferentiated conditions, cells grown in hPL showed an increase intracellular triglyceride accumulation relative to cells cultured in FBS. Following differentiation, this trend reversed somewhat, with FBS-supplemented BH isolates accumulating approximately 25 mg triglyceride/mg protein. This increase was not significantly different from commercial ASCs but was significantly higher than from FBS-supplemented HAP isolates and both hPL-supplemented groups (Figures 5(a) and 5(b)). Accumulation of neutral lipids in both noninduced control and samples treated with induction agents was assessed by Oil red O quantitation (Figure 5(c)) as well as through light micrograph images (Figure 6). The trends were observed in the relative fold increase in the Oil red O OD value of differentiated cells compared to that in their respective media controls and were similar to trends observed in triglyceride accumulation experiments. Both hPL-supplemented samples showed a significantly low fold change in Oil red O staining following differentiation. While FBS-supplemented BH isolates demonstrated the greatest fold change, followed by the FBS-HAP isolates.

### 3.6. Platelet Lysate Alters Expression of Early and Late Markers of Osteogenesis

**Figure 7:**
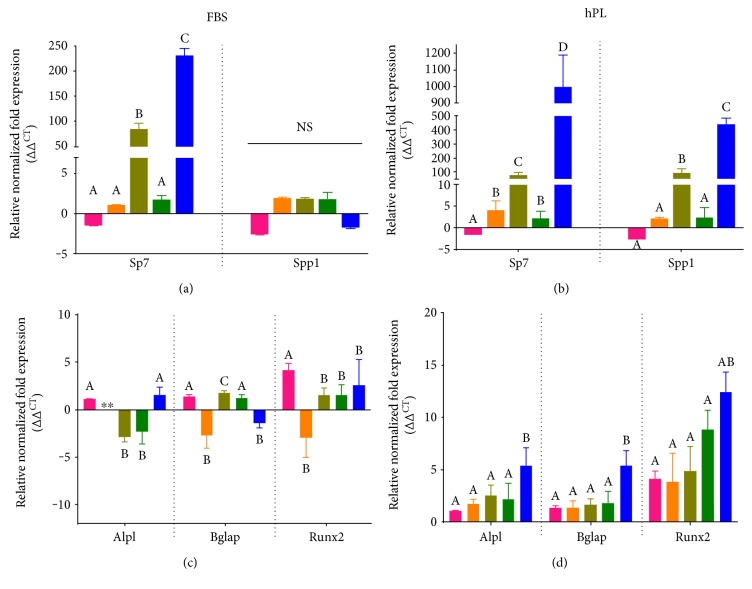
Early and late markers of osteogenesis. RNA was isolated from cells differentiated in media supplemented with 10% FBS or 10% hPL, and expression of early and late markers was measured by RT-PCR and is presented for differentiated commercial ASCs (RB-OSTEO), undifferentiated controls (HAP-CTRL and BH-CTRL), and cells cultured under osteogenic conditions (HAP-OSTEO and BH-OSTEO). All conditions were normalized to undifferentiated controls (data not shown). *Sp7* and *Spp1* expression is given for FBS (a) and hPL (b) conditions at day 7. *Alpl*, *Bglap*, and *Runx2* are presented for day 14 for FBS (c) and hPL (d). Statistically significant differences were determined by one-way ANOVA with Tukey's multiple comparisons. Data represent the mean ± SEM of three biological replicates from three donors/group. RB represents the mean ± SEM of three biological replicates from one donor from a commercial source. Significant differences between groups are designated by alphabetical letters. Any groups with the same letter are not statistically different from each other (*p* < 0.05, Tukey's MCT). A designation of ∗∗ indicates that transcript was not detected for this sample.

Isolates were induced to differentiate towards the osteogenic lineage over the course of 14 days. After one week in osteogenic induction media, cell lysates were collected for analysis by RT-PCR. Early markers of osteogenic differentiation, *SP7* and *SPP1*, were analyzed in day 7 samples and compared to those of nondifferentiated commercial ASCs (Figures 7(a) and 7(b)). For this experiment, the undifferentiated commercial ASCs, HAP, and BH groups were designated RB-CTRL (not shown on graphs but assigned a value of 1), HAP-CTRL, and BH-CTRL, respectively, while samples that have been differentiated under conditions favoring osteogenesis were designated RB-OSTEO, HAP-OSTEO, and BH-OSTEO. Significant differences in expression patterns were detected between the FBS- and hPL-supplemented groups in both the basal and differentiated conditions. Under basal conditions, FBS supplementation did not induce significant upregulation of any genes for any isolates when compared to undifferentiated commercial ASCs. For samples grown in FBS under osteogenic conditions, only *SP7* was upregulated, with an 85-fold or 230-fold increase in the HAP and BH isolates, respectively. In contrast, under basal conditions, hPL supplementation did cause significant upregulation of *SP7* in the HAP and BH isolates; however, no other significant increases were detected in any other genes for the basal hPL conditions. At day 7, the fold change for isolates grown under osteogenic conditions was even more profound, with the HAP and BH samples exhibiting a 90- and 1000-fold increase in *SP7* and a 90- and 400-fold increase in *SPP1*, respectively. After 14 days in hPL-supplemented media, only BH samples cultured under osteogenic conditions showed significant changes in expression; *ALPL* and *BGLAP* were both upregulated with a 5-fold change and *Runx2* was upregulated by 12-fold over undifferentiated commercial ASCs.

### 3.7. Alkaline Phosphatase Activity Is Modulated by Osteogenic Differentiation and Platelet Lysate Supplementation

**Figure 8:**
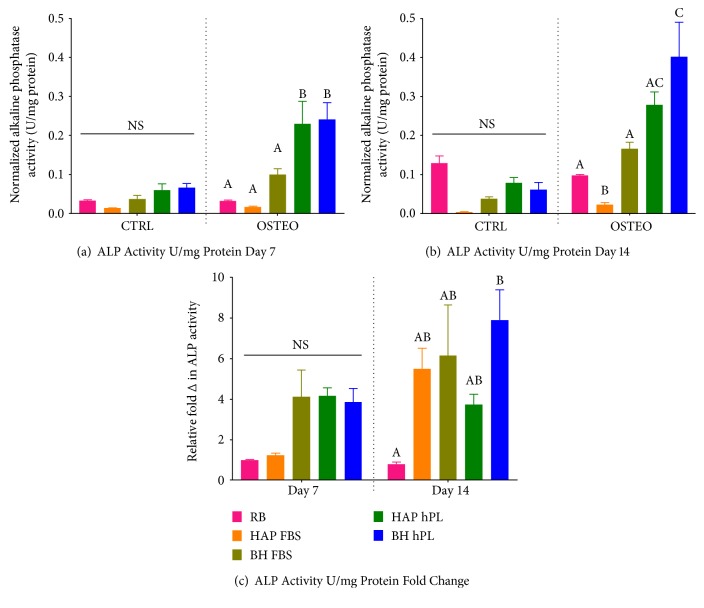
Assessment of alkaline phosphatase activity in osteogenic differentiated and control undifferentiated ASCs. ALP activity was assessed in cells under cultured control (CTRL) and osteogenic conditions (OSTEO) in media supplemented with 10% FBS or 10% hPL, then normalized to cellular protein content by BCA. Data are expressed as ALP activity (U/mg protein) in undifferentiated and differentiated cells at day 7 (a) and day 14 (b) and as fold change over control conditions following 7 and 14 days osteogenic differentiation (c). Statistically significant differences were determined by one-way ANOVA with Tukey's multiple comparisons. Data represent the mean ± SEM of three biological replicates from three donors/group. RB represents the mean ± SEM of three biological replicates from one donor from a commercial source. Significant differences between groups are designated by alphabetical letters. Any groups with the same letter are not statistically different from each other (*p* < 0.05, Tukey's MCT).

**Figure 9:**
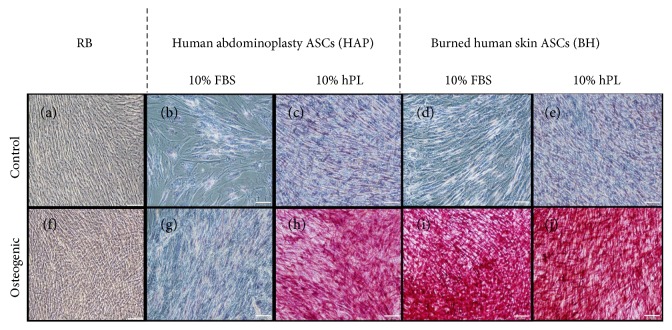
Light micrographs of Alizarin red S staining. ASCs bought from a commercial vendor (RB) were compared to ASCs derived from abdominoplasty (HAP) or burned human skin (BH) cultured in *α*MEM with 10% FBS and 10% hPL and differentiated under osteogenic conditions with identical supplements for 14 days, then stained to assess mineralization. Representative images of undifferentiated RB (a), HAP cells (b, c), and BH cells (d, e) from control media conditions and differentiated RB (f), HAP cells (g, h), and BH (i, j) are given. White scale bar represents 100 *μ*m.

As a measure of mineralization, ALP activity was measured in both differentiated and undifferentiated groups at the 7- and 14-day time points and then normalized to protein (Figures 8(a), 8(b), and 8(c)). In undifferentiated samples, no significant increase was observed in ALP activity over undifferentiated RB control, regardless of media supplement or time point. A significant increase in ALP activity for the differentiated (OSTEO) HAP-hPL and BH-hPL treatment groups was detected at the day 7 time point, compared to that for the differentiated RB, HAP-FBS, and BH-FBS groups. However, the ALP activity level was not significantly different between the HAP-hPL and BH-hPL groups at one week (Figure 8(a)). By day 14, only BH-hPL expressed higher ALP activity levels than the control and FBS-supplemented groups, while significantly higher levels of ALP were detected in the HAP-hPL group compared to those in the HAP-FBS group (Figure 8(b)). When normalizing fold changes between the two time points, the induced RB sample contained the lowest ALP activity level and the BH-hPL samples exhibited the highest hPL activity overall, while the activity induced in the HAP-FBS, BH-FBS, and HAP-hPL groups was not significantly different from either the RB or BH-hPL group (Figure 8(c)). Finally, mineralization was visualized by Alizarin red S staining (Figure 9).

## 4. Discussion

Stem cell-based treatment approaches are increasingly utilized to address a wide variety of complications ranging from pathological diseases to tissue regeneration. Mesenchymal stem cells are of particular interest to the field of regenerative medicine due to their wide array of therapeutic benefits [31–36]. The majority of published research has utilized ASCs isolated from either adipose tissue biopsies of abdominal tissue or from lipoaspirate collected during liposuction procedures [37–39]. Human lipoaspirate is also currently used for adipose tissue reconstruction procedure; the reparative effect of lipoaspirate is currently attributed to the ASCs present within the heterogeneous mixture of cells [40–43]. ASC therapy, including the expansion of autologous ASCs *in vitro*, is an excellent solution in cases where sourcing cells and tissue from patients' own body are extremely limited, especially in cases of traumatic extremity injuries that lead to extensive loss of skin, bone, or muscle [44–47]. Burn injuries, in particular, are among some of the most debilitating condition, as the patient requires extensive reparative procedures, such as grafting or skin substitute application, and the availability of autologous skin for grafting can be limited [48–51]. We have shown previously that ASCs can be sourced from burn tissue that is usually discarded after surgical excision and that these ASCs can be cryopersevered for later use [27, 52]. In this current study, we investigated the functionality of ASCs derived from discarded human burn tissue (BH) from three individual patients (*n* = 3) ranging in age from 25 to 75 years of age and ASCs from subcutaneous adipose tissue from three abdominoplasty patients (HAP; *n* = 3) of approximately 45–60 years of age. ASCs from all patients used in this study were able to differentiate into adipogenic and osteogenic lineages, corroborating our previous findings [27].

Seven-day proliferation rates of ASCs isolated from HAP and BH isolates were not statistically different when expanded in the presence of FBS. In the presence of hPL, BH cell counts were significantly higher by days 6 and 7, compared to those of the HAP isolates, indicating that the 10% hPL concentrations used in this study were not toxic or deleterious to cell proliferation. All ASCs used in this study positively expressed a panel of stem cell markers as determined by RT-PCR—CD73^+^CD90^+^CD105^+^— regardless of supplement (Supplemental Figure 1). However, this preliminary expression data did not correlate well with immunophenotypic analysis, as ASCs grown in hPL expressed low surface marker staining for CD105 and CD142, as determined by FACS analysis (Figure 3).

The safety of the stem cell administration route to a patient must be considered; therefore, we also conducted preliminary experiments to assess the impact of growth supplement on the procoagulant activity of BH and HAP isolates as well as expression of CD142 (tissue factor), an important initiator of coagulation. The number of CD142-positive cells was approximately 20% higher in the FBS-supplemented groups, as compared with that in the hPL-supplemented isolates, regardless of ASC source. However, there were large, consistent reductions in clotting times of 60–70% as assessed by thromboelastography (TEG) regardless of cell source or growth supplement (Supplemental Table 1). This suggests that both FBS and hPL-supplemented cells may increase the risk of thrombosis if administered intravenously. Currently, the majority of the clinical trials conducted have used a systemic route as the preferred mode of ASC delivery. Our results show that ASCs cause significant activation of coagulation factors and therefore suggests that intravenous (IV) administration could increase thrombotic risk in vulnerable patient populations. Although the focus of this study was not to prepare ASCs for systemic administration, IV delivery of ASCs to treat burn wounds has to be considered with a note of caution. Therefore, future research is needed to optimize ideal culture and delivery conditions that can be used for ASCs irrespective of their source and eventual use.

Though our preliminary results showed that ASCs from injured or uninjured tissue source were able to maintain some “stemness” as assessed by expression of cell surface markers, it is vital to determine the capacity of these cells to functionally differentiate into multiple cell lineages. Growing knowledge on the importance of optimizing cell growth conditions to reduce the variance attributed by media constituents has resulted in the emergence of “xeno-free” or FDA-approved synthetic alternatives [26, 53, 54]. Moreover, the risk of cells becoming primed towards a specific lineage by media constituents has been less investigated. Contrary to our preliminary expression data, FACS analysis demonstrated that cells grown in hPL exhibited low expression of CD105, which, in ASCs, is associated with increased osteogenic gene expression, indicating that hPL may select for a more osteogenic lineage [55]. Therefore, we sought to determine the influence of DMEM or *α*-MEM media supplemented with 10% hPL on ASC multipotency, in comparison to that of the traditional basal media supplemented with 10% FBS. It is important to note that a commercial ASC isolate was used for normalization in this study. We chose to add this isolate because it was a commercial sample, derived from a healthy, nonobese individual, as we are not able to make any assumptions regarding the generalizability of our abdominoplasty-derived ASCs to a healthy population.

A recent study showed ASCs cultured in the presence of hPL express pluripotency genes and, most importantly, osteogenic-, adipogenic-, and chondrogenic-associated markers [56]. When we analyzed that basal level expression of transcripts specific to the adipogenic lineage, both BH and HAP ASCs, expressed a modest level of *PPARG* in basal media supplemented with either FBS or hPL. The inherent nature of ASCs to express *PPARG*, a member of the nuclear receptor superfamily responsible for promoting early transcription involved in adipogenesis, is attributed to their origin in adipose tissue [57, 58]. However, commercial ASCs (RB) obtained from a healthy individual showed higher basal expression of *PPARG* before any induction, indicating a possible influence of initial culture media and source of ASCs. The classical way of determining an ASC isolate's ability to differentiate into mature adipocytes involves the addition of specific induction agents [59]. It is well known that adipogenesis follows a well-organized, glucose-regulated signaling pathway under the control of CCAAT/enhancer-binding protein alpha (*C/EBPα*) and *PPARG* [60]. During this process, PPARG heterodimerizes with another nuclear receptor, the retinoid X receptor-*α* (RXR*α*) to bind DNA and promote the transcription of adipocyte-specific genes such as adiponectin and leptin [61]. As such, we chose to evaluate the expression of *PPARG*, as well as adipocyte-specific genes, and *GLUT4*, an insulin-regulated glucose transporter. In this study, we noticed strong influences of the supplements on expression of these genes. In particular, during differentiation, hPL strongly downregulated expression of *ADIPOQ*, a protein that is generally strongly upregulated during adipogenesis and that is involved in insulin sensitization, glucose regulation, and the utilization of fatty acids. This effect was not seen in the HAP and BH samples cultured in FBS with adipogenic conditions, as adiponectin was expressed at levels that were similar to the differentiated commercial ASCs. Interestingly, expression of *LEP*, a hormone involved in energy utilization, was significantly and strongly upregulated only in the HAP isolates differentiated in the presence of FBS. When differentiated in the presence of FBS, triglyceride (TG) levels were significantly higher in ASCs from burned tissue compared to those derived from abdominoplasty. As shown in Figure 3, significant triglyceride levels were detected in the basal hPL conditions, a finding that correlated with the increased observation of Oil red O accumulation visible in the light micrographs in Figure 4. However, no significant induction of triglyceride accumulation was detected when ASCs were differentiated in the presence of hPL. Collectively, the observed results indicate that hPL may suppress terminal adipogenic differentiation of ASCs; the factors that are responsible for this observation remain to be explored.

Another aspect of ASC functionality is their ability to differentiate towards an osteogenic lineage. It is well documented that ASCs under the influence of inducers such as BMP-2, insulin, and dexamethasone will transdifferentiate to osteoblast-like cell [60]. However, it is less apparent whether there are donors and source-dependent or culture supplement-dependent differences that can influence osteogenic differentiation. It has been reported that MSCs expanded using commercial grade hPL produced under good manufacturing practice (GMP) exhibit spontaneous activation of zinc finger transcription factor (*SP7)* as well as activation of *RUNX2*, a master osteogenic transcription factor responsible for mature osteoblast formation. *RUNX2* is central to the osteogenic switch and responsible for the downstream signaling cascade involved in this differentiation process [56]. In this study, we observed early commitment towards osteogenesis in ASCs derived from both burned debrided tissue and abdominoplasty when grown in hPL-supplemented media, as compared to FBS supplementation. When maintained or differentiated in the presence of FBS, only cells in the osteogenic induction media exhibited increases in markers of osteogenesis and only for the early marker, *SP7*. In contrast, cells grown in hPL showed a significant increase in the basal level expression of *SP7*; an effect was seen in both HAP and BH cells compared with that in the commercial RB ASCs. Furthermore, expression of *Spp1* (osteopontin), a protein involved in bone mineralization, was significantly upregulated in ASCs differentiated in the presence of hPL at day 7. After two weeks in hPL-supplemented osteogenic media, only the ASCs from burned debrided tissue exhibited upregulation in the late markers of osteogenesis.

Much of the research using hPL as a culture supplement has focused on the abundant presence of mitogenic growth factors (e.g., PDGF, TGF EGF, FGF, and VEGF) in hPL [25, 62–64]. A number of other proteins are present in hPL, including insulin, OPN, and IGFBP, which may affect “stemness” in MSCs. Specifically, protein array analysis of the hPL used in this study indicates the presence of several types of IGFBPs at >100 ng/ml and also detectible amounts of OPN. IGFBP-3 along with IGF-1 has been shown to encourage new bone formation [65] and functions upstream with *Runx2* to induce bone mineralization [66]. IGFBP-3 and OPN together with exogenously supplemented BMP may stimulate ASCs to differentiate towards an osteogenic phenotype.

FBS is considered a “gold standard” to which all new media supplements would be compared; however, there is limited availability of characterization data for commercial FBS. While proteomic analysis has identified proteins like IGF and IGFBP and TFG*β* in FBS, there appears to be significant lot to lot variability [67]. The lack of full characterization of FBS may make a direct comparison of the contents of the two media supplements impossible at this time. Furthermore, testing the individual components of hPL on ASC isolates was beyond the scope of this study; however, a synergistic effect was apparent in both BH- and HAP-derived ASCs that were induced with osteogenic stimuli. All of the osteogenic transcription factors analyzed (*ALPL*, *RUNX2*, and *BGLAP*) were significantly upregulated in comparison to cells grown in basal media supplemented with FBS. Among the ASCs propagated in hPL, cells derived from BH exhibited the highest level of *ALPL* expression, indicating ASCs from burn patients may have higher sensitivity to external stimuli. This finding is supported by the decreased surface marker expression of CD105 in both HAP and BH (31% and 48% reduction, resp.) cells cultured in the presence of hPL. This observed increase in expression levels of osteogenic markers in hPL-supplemented ASCs, as well as the finding that cells cultured in hPL have low basal surface marker expression of CD105, indicates an increased responsiveness in these conditions; it confirms that factors in hPL influence ASC's propensity towards osteogenesis. This effect may be amplified in the setting of injury, as evidenced by the enhanced reactivity of BH cells to osteogenic stimuli.

To this end, we observed that hPL as a media supplement significantly influences osteogenesis, although it is noteworthy that ASCs from burned tissue grown in FBS media were less sensitive to osteoinduction and showed downregulation of *ALPL* expression in comparison to ASCs from a healthy individual (RB). The ALP activity levels further confirmed our observation, wherein ALP deposition was highest in BH ASCs. Though the observed fold change in ALP activity after induction was lowest in commercial RB ASCs, the HAP cells showed the least amount of protein activity postinduction. We speculate that ASCs from HAPs may have been influenced by altered glucose metabolism, as shown elsewhere by other investigators [68]. We are not able to confirm the present or former metabolic status of the samples of the HAP cells used in this study; however, HAP ASCs were still responsive to external osteogenic stimuli, even in FBS growth condition. Collectively, our study shows the following: (i) while FBS supports adipogenic differentiation in both groups, hPL may enhance basal lipid accumulation while hindering terminal adipogenesis, (ii) there is a higher inherent level of osteogenic transcripts in ASCs from burn samples, which is highly sensitive to external stimuli and to the factors within hPL, (iii) ASCs derived from abdominoplasty are less sensitive to osteogenic stimulus in comparison to BH ASCs but are not significantly different from the commercial ASCs from a healthy individual, and (iv) there are differences among ASCs that are dependent upon the donor, as evidenced by individual proliferation rates and CD142 surface staining (Supplemental Figure 2 and Supplemental Figure 3) and source that may influence differentiation potential. Therefore, expansion of ASCs in hPL has to be considered with caution. Although hPL is “xeno-free”, the effects on MSC phenotype warrant extensive, long-term investigation.

## 5. Conclusion

The potential therapeutic benefits of ASCs are many, and extensive research has been conducted in the past decade to understand their cellular and molecular functionality. The next steps in development include identification of optimal sources and definition of expansion methods and optimal delivery methods to preserve ASCs “stemness and functionality” for treatment purposes as well as safety. Although the ASC niche is the perivascular region of adipose tissue stroma, we observed functional differences from different donor sources. ASCs isolated from discarded burn adipose tissue differed from ASCs derived from abdominoplasty samples. Specifically, BH ASCs contained increased inducible levels of factors that favored differentiation towards an osteogenic rather than an adipogenic phenotype. Further, when BH ASCs expanded in human platelet lysate, an extensively investigated xeno-free alternative to expand MSCs for translational purposes had a significant effect on its functionality irrespective of cell source. The primary goal of this study was to evaluate hPL as a nutrient source in cell expansion conditions prior to the topical use of ASCs in treating burn wounds. Collectively, this study shows that both ASCs derived from uninjured (abdominoplasty) and injured adipose tissue (debrided burn tissue) exhibited an altered immunophenotype under the influence hPL, specifically characterized by low surface expression of CD105 and CD142 and preferentially differentiated into osteogenic lineage. Altogether, our results indicate that culture with hPL may prime stem cells towards an osteogenic lineage and must be carefully considered before use in clinically relevant samples.

## Supplementary Material

Supplemental Figure 1. Total RNA was collected from cell lysates from isolates expanded in 10% FBS or hPL just prior to differentiation (D0). Cells were analyzed for the genes encoding CD73, CD90, and CD105. Data are presented as fold change over *GAPDH* (Target C_T_/*GAPDH*). Data represent the mean ± SEM of three biological replicates from at least three isolates. Treatment groups with the same letter are not significantly different (*p* < 0.05). Supplemental Figure 2. Proliferation of individual cell isolates in FBS or hPL. Cells at P2 or P3 were seeded in a microplate at 2000 cells/well in media supplemented with FBS (A) or hPL (B) and allowed to proliferate for up to 7 days, with time points collected every 24hrs. Data represents the mean ± SEM of three biological replicates from three donors/group. RB (C) represents the mean ± SEM of three biological replicates from one donor from a commercial source. Supplemental Table 1. ASCs were evaluated for expression of tissue factor (CD142) by flow cytometry and for pro-coagulant activity. Data are expressed as percent of parent positive for CD142 and % R time reduction for TEG.
