# Supplementary material for: Tissue Source and Cell Expansion Condition Influence Phenotypic Changes of Adipose-Derived Stem Cells
Source: Stem Cells Int. 2017 Aug 23;2017:7108458. doi: 10.1155/2017/7108458 (PMC5613713; doi:10.1155/2017/7108458)
Supplement: Supplementary file 1 — Supplemental Figure 1. Total RNA was collected from cell lysates from isolates expanded in 10% FBS or hPL just prior to differentiation (D0). Cells were analyzed for the genes encoding CD73, CD90, and CD105. Data are presented as fold change over GAPDH (Target CT/GAPDH). Data represent the mean ± SEM of three biological replicates from at least three isolates. Treatment groups with the same letter are not significantly different (p < 0.05). Supplemental Figure 2. Proliferation of individual cell isolates in FBS or hPL. Cells at P2 or P3 were seeded in a microplate at 2000 cells/well in media supplemented with FBS (A) or hPL (B) and allowed to proliferate for up to 7 days, with time points collected every 24hrs. Data represents the mean ± SEM of three biological replicates from three donors/group. RB (C) represents the mean ± SEM of three biological replicates from one donor from a commercial source. Supplemental Table 1. ASCs were evaluated for expression of tissue factor (CD142) by flow cytometry and for pro-coagulant activity. Data are expressed as percent of parent positive for CD142 and % R time reduction for TEG. [file 7108458.f1.pdf]

Supplemental Figure 1

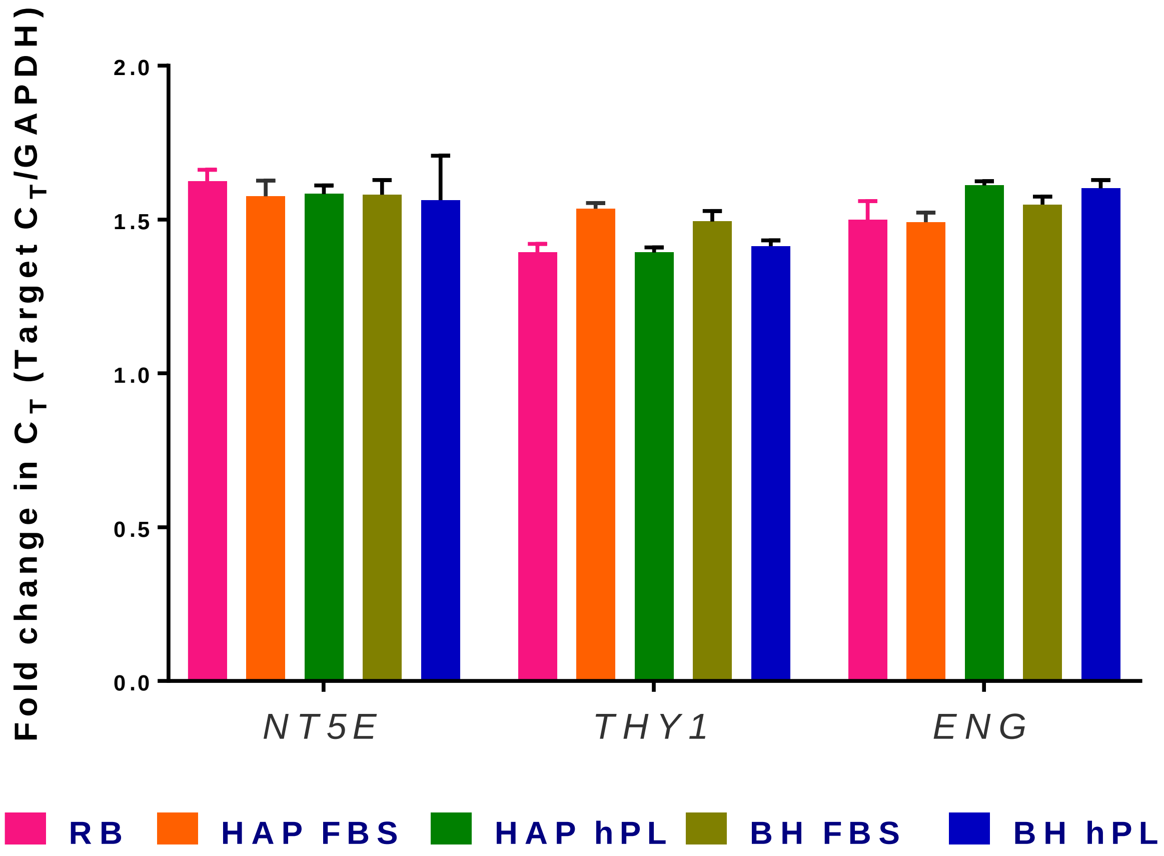

Supplemental Figure 2

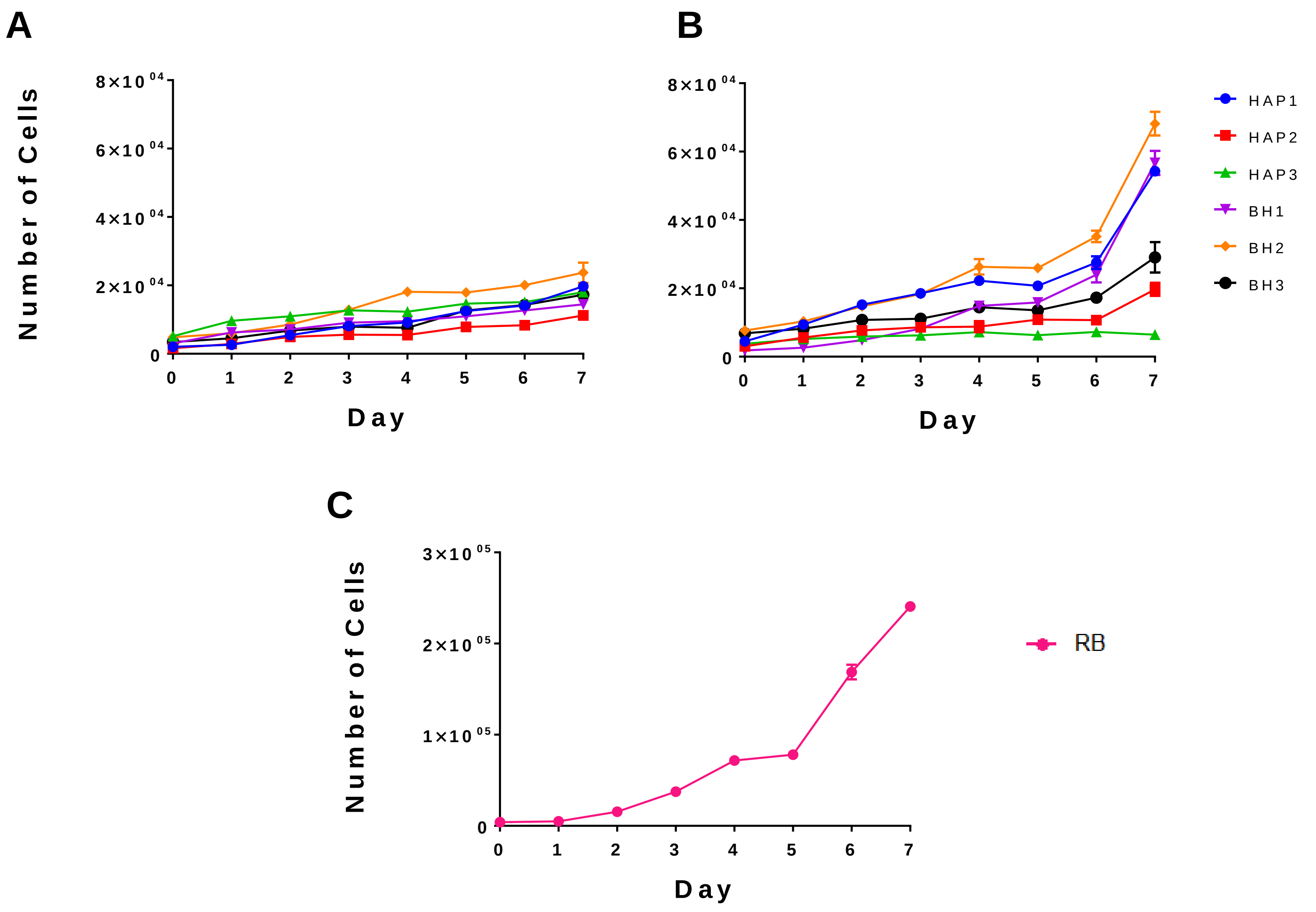

Supplemental Table 1

Supplement	Isolate	CD142 %Parent	% R Time Reduction
RB High Performance Media	hAD-RB00088	94.6	61.2
FBS	HAP1	85.9	71.9
	HAP2	87.5	70.1
	BH1	56.4	67.9
	BH2	78.3	64.3
hPL	HAP1	52.2	64
	HAP2	67.1	59.7
	BH1	69.8	59.6
	BH2	67.6	59.5
