# Supplementary figures and images for: Tissue Source and Cell Expansion Condition Influence Phenotypic Changes of Adipose-Derived Stem Cells
Source: Stem Cells Int. 2017 Aug 23;2017:7108458. doi: 10.1155/2017/7108458 (PMC5613713; doi:10.1155/2017/7108458)

## Slide 1
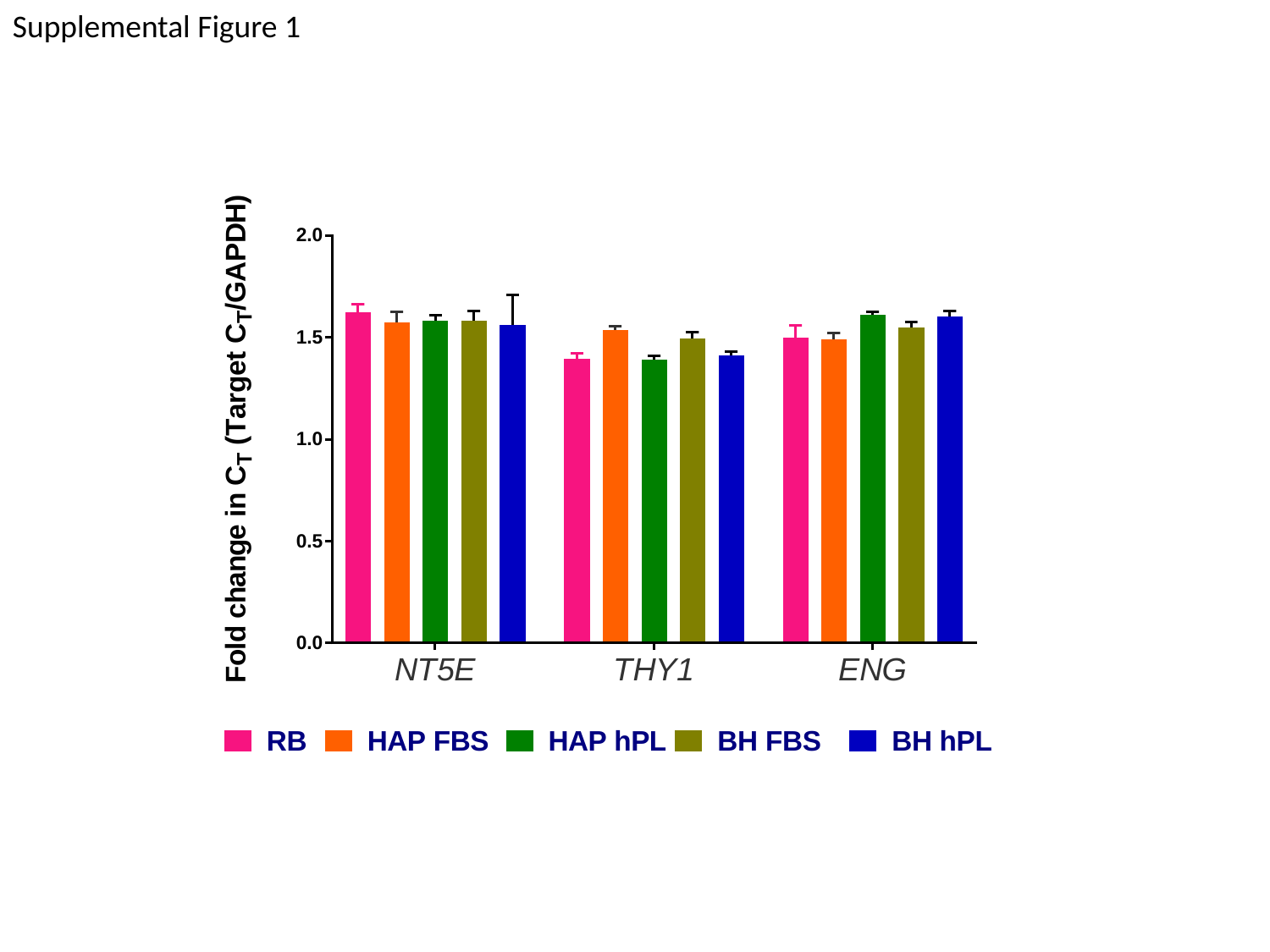

Supplemental Figure 1

Supplement: Supplementary file 2 [file 7108458.f2.pptx]

## Slide 1
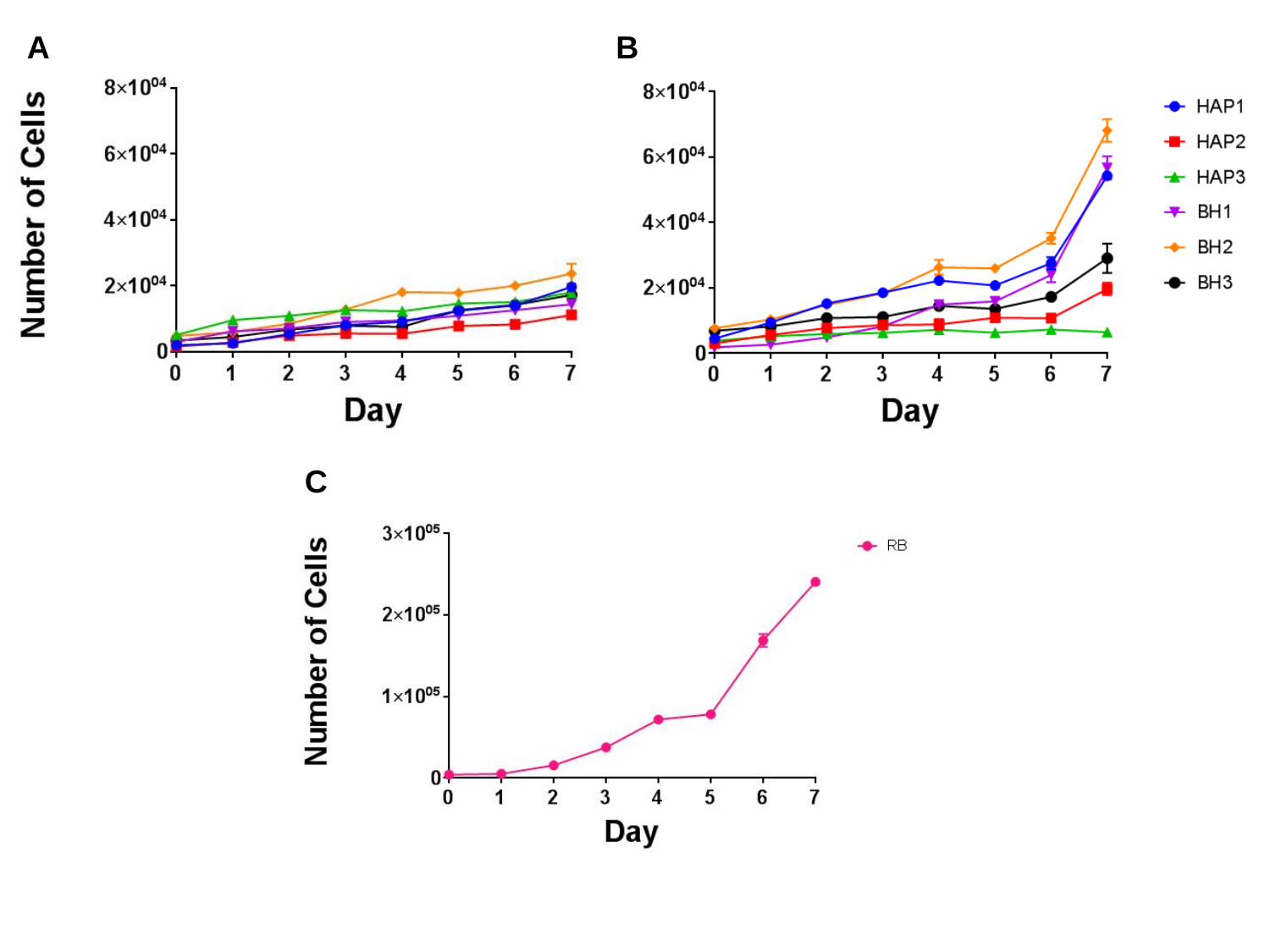

A
B
C

Supplement: Supplementary file 3 [file 7108458.f3.pptx]
