# Supplementary material for: Tissue Source and Cell Expansion Condition Influence Phenotypic Changes of Adipose-Derived Stem Cells
Source: Stem Cells Int. 2017 Aug 23;2017:7108458. doi: 10.1155/2017/7108458 (PMC5613713; doi:10.1155/2017/7108458)
Supplement: Supplementary file 4 [file 7108458.f4.pptx]

## Slide 1
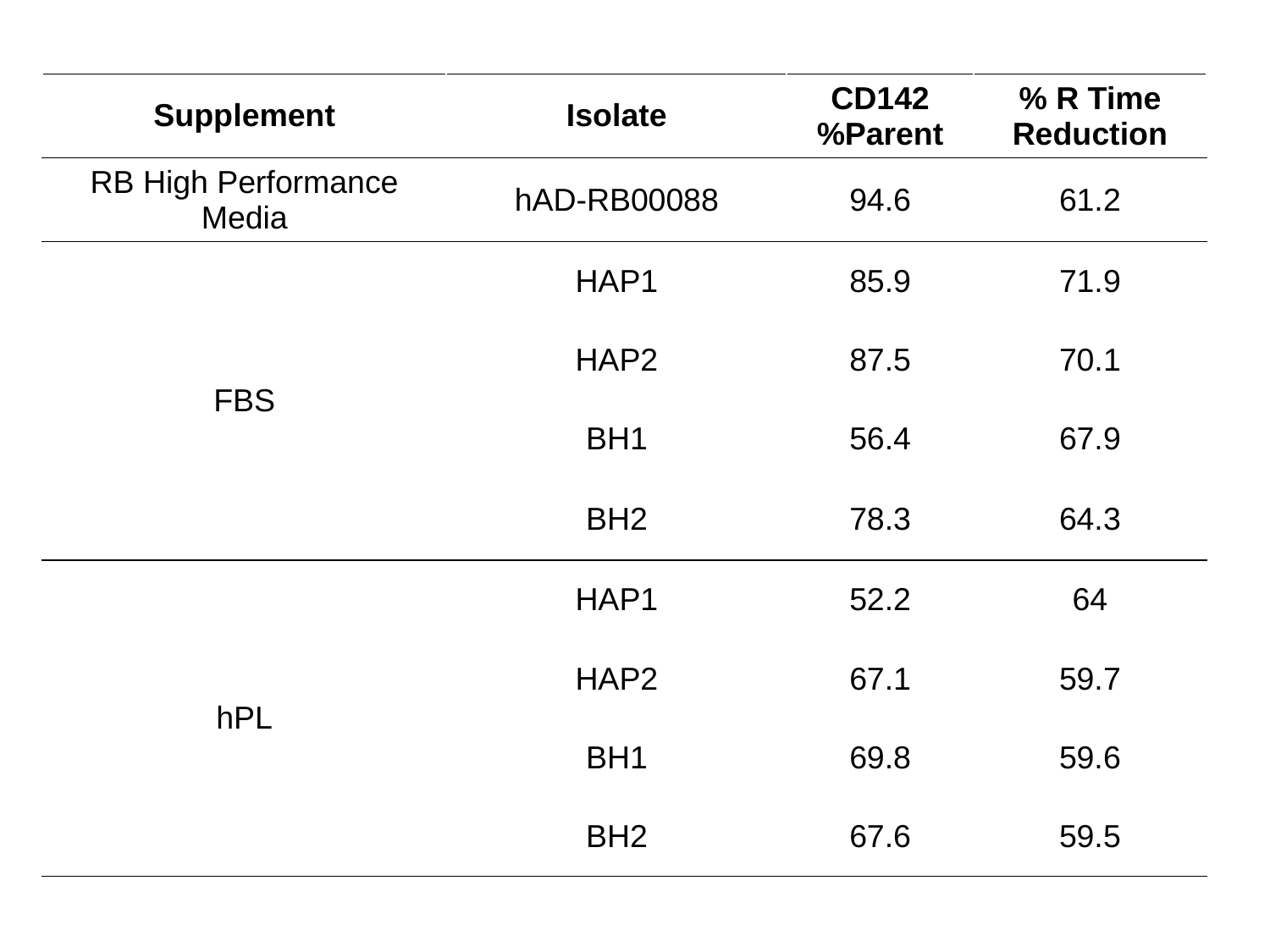

| Supplement | Isolate | CD142 %Parent | % R Time Reduction |
| --- | --- | --- | --- |
| RB High Performance Media | hAD-RB00088 | 94.6 | 61.2 |
| FBS | HAP1 | 85.9 | 71.9 |
| | HAP2 | 87.5 | 70.1 |
| | BH1 | 56.4 | 67.9 |
| | BH2 | 78.3 | 64.3 |
| hPL | HAP1 | 52.2 | 64 |
| | HAP2 | 67.1 | 59.7 |
| | BH1 | 69.8 | 59.6 |
| | BH2 | 67.6 | 59.5 |
